# Serum vaspin level as a predictive indicator in the amelioration of fatty liver and metabolic disturbance in patients with severe obesity after laparoscopic vertical banded gastroplasty

**DOI:** 10.1097/MD.0000000000007498

**Published:** 2017-07-28

**Authors:** Yong Wang, Zong-Fan Yu, Yun-Sheng Cheng, Ben-Li Jia, Gang Yu, Xiao-Qiang Yin, Yang Wang

**Affiliations:** Department of General Surgery, The Second Hospital of Anhui Medical University, Hefei, Anhui Province, People's Republic of China.

**Keywords:** fatty liver, metabolic disturbance, predictive value, serum vaspin level, severe obese patients

## Abstract

**Background:**

This study is all about predicting the value of serum vaspin level in the amelioration of fatty liver and metabolic disturbance in patients with severe obesity after laparoscopic vertical banded gastroplasty (LVBG).

**Methods:**

A total of 164 patients (from January 2012 to May 2015) with severe obesity were chosen and performed LVBG. Enzyme-linked immunosorbent assay was performed to detect the serum vaspin level. The patients were given a biochemical automatic analyzer to measure the biochemical indicators. Homeostasis model assessment (HOMA) helps in the calculation of fasting insulin level (FINS) and insulin resistance (IR). The changes in fatty liver were examined by computed tomography (CT). Receiver operating characteristic curve is used to increase the predictive value of serum vaspin level in the amelioration of liver function and disturbances in the metabolism.

**Results:**

Weight, BMI, waist circumference, serum vaspin level, and triglyceride (TG) decreased, but CT value of liver increased at 4th, 7th, and 12th month after surgery. After the 7th and 12th month period of surgery, the alanine aminotransferase, aspartate aminotransferase, FINS, and HOMA-IR reduced in the patients (*P* <.005). The area under ROC curve (AUC) is about 0.871 ± 0.031 with 95%CI of 0.810–0.931 (*P* <.001). The sensitivity, specificity, and accuracy of serum vaspin level ≤0.9 were 87.80%, 78.05%, and 83.28%, respectively. BMI, FINS, and serum vaspin level ≤0.9 were the influencing factors of the amelioration of fatty liver and metabolic disturbance.

**Conclusion:**

This study proves that the serum vaspin level serves as a predictive indicator in the amelioration of fatty liver and metabolic disturbance in patients with severe obesity after LVBG.

## 1 Introduction

Obesity is now one of the most increasingly serious threat to global health, which affects more than 500 million adults worldwide.^[1,2]^ The more important alarming fact is the incidence of severe obesity body mass index (BMI) ≥35 kg/m^2^.^[3,4]^ Severe obesity is highly associated with some chronic diseases like cardiovascular disease, diabetes, and carcinoma, leading to high mortality and morbidity.^[5–7]^ It is also proved that severe obesity contributes to fatty liver and metabolic disturbances; several factors, including insulin sensitivity, blood pressure, as well as plasma lipid profile, are associated with excessive accumulation of fat in visceral adipose tissue (VAT), resulting in dyslipidemia, damaged glucose tolerance, and hypertension.^[8,9]^ According to the study, it is reported that adipocytokines secreted from VAT have regulatory effects on insulin sensitivity, energy homeostasis, inflammation, vascular functions as well as lipid metabolism.^[10]^ Presently, bariatric surgeries have been generally considered as the most effective treatment for sustained and durable weight loss in patients with severe obesity due to its preventability and therapeutic effect.^[11,12]^

Vaspin (VAT-derived serine proteinase inhibitor), is a significant and relatively novel adipocytokine with regulation functions in lipid and glucose metabolisms, has been found to be exclusively present in the VAT with obesity of Otsuka Long-Evans Tokushima Fatty rats.^[13–15]^ Then later all the researches have proved that vaspin is present in human tissues, such as skin, stomach, pancreas, liver, and adipose tissue.^[16–18]^ It has been proved that serum vaspin levels are positively correlated to metabolic syndrome parameters.^[19]^ As a beneficiary adipocytokine, vaspin acts on obesity-related metabolic dysfunction through genetic manipulation and exerts insulin-sensitizing effects and anti-inflammatory properties to improve insulin sensitivity and alleviate endoplasmic reticulum stress in liver.^[20,21]^ Aktas et al^[22]^ proved that vaspin serum levels are independent predictive value for liver fibrosis with nonalcoholic fatty liver diseases in patients. However, the patients with severe obesity with serum vaspin levels who had surgery are not yet fully explored. This study proves about the predictive value of serum vaspin level for the amelioration of fatty liver and metabolic disturbance in patients with severe obesity after treating with laparoscopic vertical banded gastroplasty (LVBG).

## 2 Materials and methods

### 2.1 Ethical statement

This study was approved by the Ethical Committee of The Second Hospital of Anhui Medical University and Jiangsu Province Hospital. All the patients included in this study signed the informed consents to be involved in this research

### 2.2 Study subjects

From January 2012 to May 2015, 164 patients with severe obesity were recruited in this study from The Second Hospital of Anhui Medical University and Jiangsu Province Hospital (91 males and 73 females, mean age: 28.12 ± 6.46 years, Quetelet index (BMI) ≥35 kg/m^2^). BMI of patients ranged from 37.03 to 52.69 kg/m^2^. The number of daily steps of patients in a year were recorded by Yamax pedometer (sw-701), and mean steps/day was measured. According to the standard of physical activity (PA)^[23,24]^: 49 case of ≥10,000 steps/day and 115 cases of <10,000 steps/day. Clinical evaluation was performed on patients like physical examination (weight, height, and waist circumference for the calculation of BMI) and relevant biochemical detection by professional doctors to rule out the secondary obesity, followed by other evaluation tests and they are prepared for the operation by the surgeons. In the mornings the day before the surgery, the fasting venous blood of all subjects was obtained, and the serum was separated after centrifugation and stored for further uses. The exclusion criteria includes: the patients with severe heart, liver, or renal dysfunction; the patients with diseases related to abnormalities of glucose and lipid metabolisms, such as diabetic ketoacidosis (DKA), pituitary damage, adrenal gland injury, thyroid damage, and secondary pancreatic injury; the patients with transient hyperglycemia caused by acute infection, trauma, or other stress.

### 2.3 Diagnostic criteria for severe obesity

Quetelet's index (BMI) is a type of measure of fatness performed on adults whose physical developments were relatively stable. BMI = weight (kg)/[height (m)]^2^. Generally, a man with BMI >25 kg/m^2^, and a woman with BMI >24 kg/m^2^ are known as overweight persons. According to research results in Shanghai, 15- to 19-year-old adolescents with BMI between 18 and 22 kg/m^2^ were considered as normal weight, with BMI ≥22 kg/m^2^ were considered as overweight, and with BMI ≥24 kg/m^2^ were considered as obese patients. Adults more than 20 years old with BMI between 20 and 24 kg/m^2^ were considered as normal weight, with BMI ≥24 kg/m^2^ were considered as overweight, and with BMI ≥26 kg/m^2^ were considered as obese patients. According to the World Health Organization, the BMI classification in 2014 confirms that, BMI between 25 and 30 kg/m^2^ are consider as overweight. The obese patients are subclassified into 3 types: pre-obese or class I obesity (BMI 30, 35 kg/m^2^), severe or class II obesity (BMI 35–40 kg/m^2^), and extreme or class III obesity (BMI ≥40 kg/m^2^). The value of BMI for the diagnostic criteria for severe obesity in this research is ≥40 kg/m^2^.^[25]^

### 2.4 Operation method

After general anesthesia is given, the patient is placed on the table in a supine or lithotomy position. After the pneumoperitoneum was established, 4–5 holes were opened, where it includes the peephole in the upper middle abdomen, the auxiliary operating hole at 5 cm under the xiphoid process, and 2 main operating holes on the left and right midclavicular lines in the upper abdomen. Another auxiliary operating hole (10 mm) could be opened on the anterior axillary line in the left mid-abdomen if necessary. The ultrasonic knife is used to dissociate the lesser curvature below the cardiac and gastric fundus. Then, fenestration of anterior and posterior wall of stomach was conducted at 6–7 cm below the cardia (near the lesser curvature) using a 21-mm circular stapler. The insulation of the upper lesser curvature from the gastric window directing to the left side of the lesser curvature of cadia is done by using the 60-mm endovascular gastrointestinal anastomosis (Endo GIA) stapler. The band of gastric pouch was formed at the gastric window using a mesh the tube drainage was set in the operating area for fasting the venous blood of all patients and obtained at 1st, 4th, 7th, and 12th month after surgery, and then the serum was separated after centrifugation and stored at –20°C for future use.

### 2.5 Diagnosis and evaluation of fatty liver

For this research before the surgery was started, 164 patients were diagnosed as fatty liver by B-mode ultrasonography. The diagnostic evidences were as follows: the near-field of liver is diffusely hyperechoic with higher echo intensity than spleen; the far-field spot is in low density accompanied by echo attenuation; and the liver is mildly or moderately enlarged with blunted leading edges. The patients were chosen on the basis of no drinking history or weekly based alcohol consumption about less than 40 g was found; negative markers of vertical transmission of hepatitis B virus (HBV) infection were detected, which were consistent with the diagnostic criteria for nonalcoholic fatty liver. Computed tomography (CT) examination of the liver was performed by the specialists using a CT scanner (Picker PQ5000, Marconi Medical Systems, Cleveland, OH). CT values of 4 lobes of liver were examined and the mean CT value of the entire liver was used for comparison. The CT diagnostic criteria of fatty liver were as follows: hepatic density decreased and CT value of the liver was lower than that of spleen (liver/spleen CT value ratio ≤1).

### 2.6 Enzyme-linked immunosorbent assay

The venous blood of all patients with limosis was collected at the 1st, 4th, 7th, and 12th month after examination on admission and surgery. The collected blood specimens from the patients were placed in a refrigerator for about an hour at 4°C with an angle of 45°–60°. Serum specimens were obtained by low-speed centrifugation at 3000 rpm/min for 5 min and stored at –20°C. The vaspin level was detected using an enzyme-linked immunosorbent assay (ELISA) kit (Bender Medsystems Gmbh, Vienna, Austria) according to the manufacture's instruction, and the optical density value was then measured at 450 nm using a microplate reader. This experiment was repeated 3 times to obtain the mean value.

### 2.7 Detection of biochemical indicators

During the detection of biochemical indicators, the fasting venous blood was obtained from the patients during the time of admission and also at 1st, 4th, 7th, and 12th month after surgery. A HITACH 17170A biochemical automatic analyzer (Hitachi Co. Ltd, Tokyo, Japan) was applied to detect the levels of fasting blood glucose (FPG), fasting blood lipid (FPL, including total cholesterol [TC], triglyceride [TG], high-density lipoprotein [HDL], and low-density lipoprotein [LDL]), liver enzyme levels (alanine aminotransferase [ALT], aspartate aminotransferase [AST], and γ-glutamyl transpeptidase [γ-GT]), and serum uric acid (SUA). The operation was repeated 3 times to obtain the mean value.

### 2.8 Homeostasis model assessment of insulin resistance (HOMA-IR) and β-cell function (HOMA-β)

This is a different type of processes where the insulin resistance and pancreatic β-cell function were assessed by the homeostasis model assessment (HOMA). The β-cell function in response to glucose loading was assessed using the modified β-cell function index (MBCI). HOMA-insulin resistance (IR) = In [(FINS×FBG)/22.5]. MBCI = (FINS×FBG)/(2 hour PG + 1 hour PG – 2FPG), where FINS is fasting insulin level; FBG  is fasting blood glucose; 1 hour PG  is 1-hour plasma glucose during oral glucose tolerance test (OGTT); and 2 hour PG  is 2-hour plasma glucose during OGTT.

### 2.9 Statistical analysis

The data collected in this research were analyzed by SPSS version 21.0 (SPSS Inc., Chicago, IL) and the measurement data were expressed as mean ± standard deviation (SD). Comparisons between preoperative and postoperative data in normal distribution were analyzed using paired *t*-test. Receiver operating characteristic (ROC) curve was drawn to evaluate the predictive value of serum vaspin level in amelioration of liver function and metabolic disturbance. Logistic regression analysis was exhibited for factors that influence the amelioration of fatty liver and metabolic disturbance in patients with severe obesity after surgery. *P* <.05 indicates the statistical significance of serum vaspin level.

## 3 Results

### 3.1 Weight, BMI, and waist circumference of patients before and after surgery

Compared with the preoperative data, no significant changes were found in the weight, BMI, and waist circumference of patients at first month after surgery (*P* >.05), but decreases in the weight, BMI, and waist circumference were observed at fourth month later (*P* <.05). At 12th month after surgery, the weight, BMI, and waist circumference of patients reduced by 31.26 ± 14.72 kg, 10.96 ± 5.34 kg/m^2^, and 20.24 ± 3.36 cm on average, respectively (Table 1).

**Table 1:**
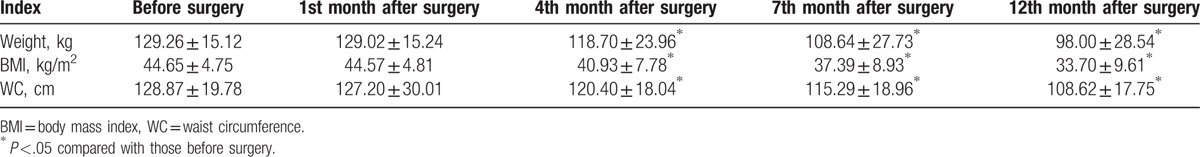
Weight, BMI and WC of patients with severe obesity before and after surgery.

### 3.2 Decreases in serum vaspin level at 4th, 7th, and 12th month after surgery

**Figure 1:**
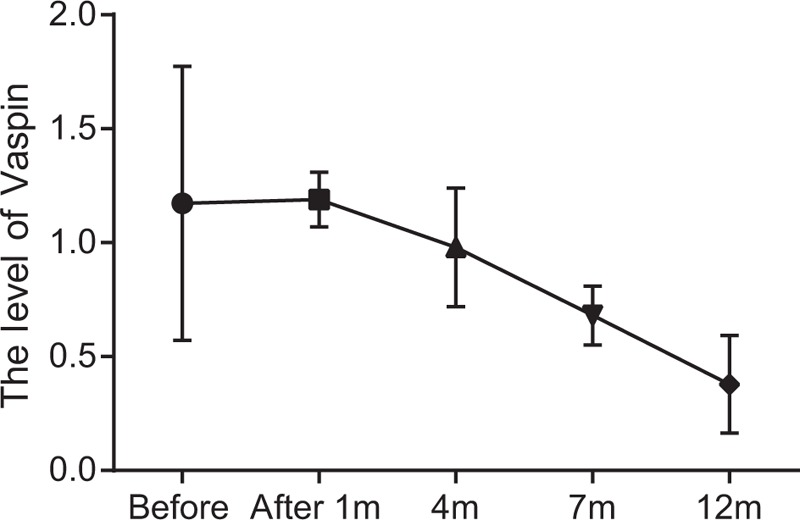
Changes in serum vaspin level before and after surgery detected by ELISA. Note: ∗, compared with the levels before surgery and 1 month after surgery, *P* <.05. ELISA = enzyme linked immunosorbent assay, m = month.

The serum vaspin levels of the patients were detected by ELISA before surgery and at 1st, 4th, 7th, and 12th month after surgery. And the serum vaspin level was gradually decreased at 4th, 7^th^, and 12th month after LVBG (*P* <.05) (Fig. 1).

### 3.3 Reduction of serum TG level at 4th, 7th, and 12th month after surgery

The details compared with data after and before surgery there are no obvious differences were presented in serum TC, LDL, HDL, and SUA levels (*P* >.05), but the serum TG level was gradually reduced at 4th, 7th, and 12th month after surgery (*P* <.05) (Table 2).

**Table 2:**
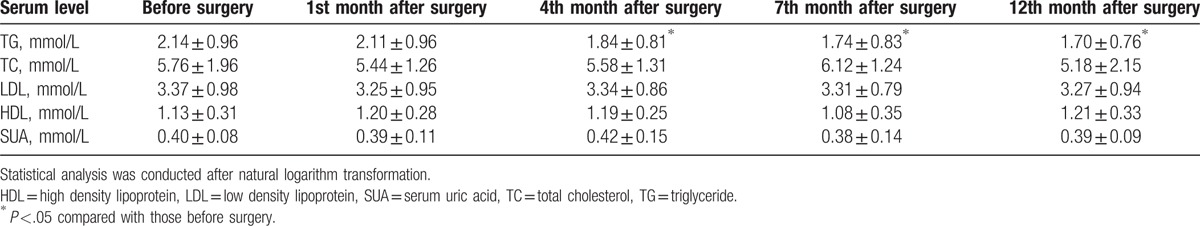
Serum TG, TC, LDL, HDL and SUA levels in patients with severe obesity before and after surgery.

### 3.4 The amelioration of fatty liver at 4th, 7th, and 12th month after surgery

Among the 164 patients, 145 patients were found to suffer from at least 1 liver enzyme abnormality that was 2 times lower than the upper limit of normal value. No significant difference was exhibited in serum γ-GT level before and after surgery (*P* >.05). Serum ALT and AST levels had no obvious change at 1st and 4th month after surgery, but decreased significantly at 7th and 12th month (*P* <.05). The mean CT value of liver at 4th, 7th, and 12th month after surgery were remarkably higher than that before surgery (*P* <.05) (Table 3).

**Table 3:**
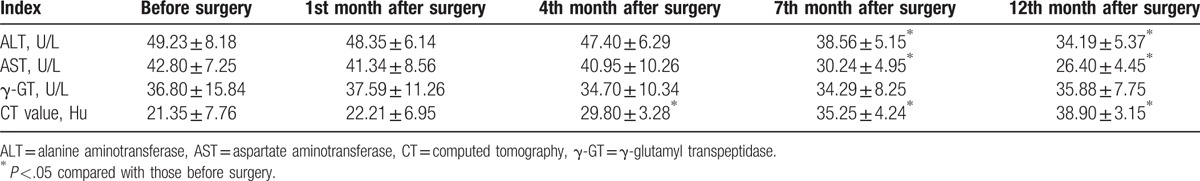
ALT, AST, γ-GT, and CT value of liver in patients with severe obesity before and after surgery.

### 3.5 Decreases in FINS and HOMA-IR at 7th and 12th month after surgery

At the fourth month after surgery, FINS and HOMA IR decreased in patients with severe obesity, and significantly decreased at the seventh month (*P* <.05) (Table 4).

**Table 4:**
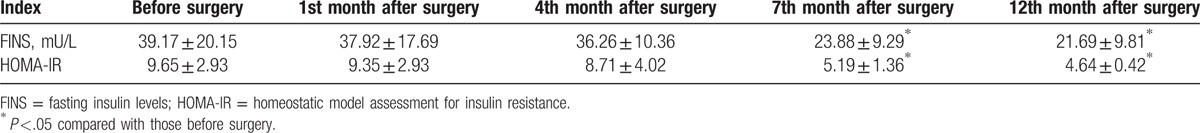
FINS and HOMA-IR in patients with severe obesity before and after surgery.

### 3.6 ROC curve analysis

**Figure 2:**
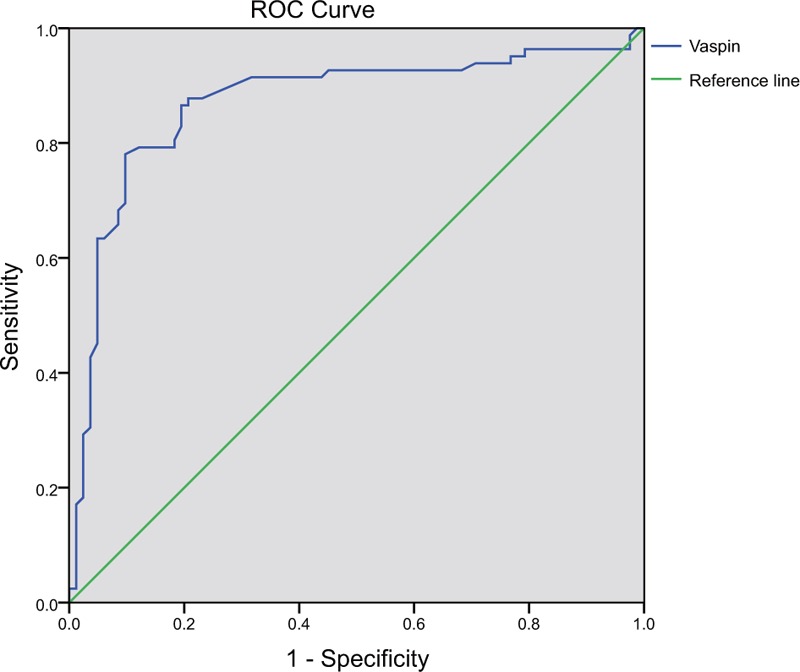
ROC curve of the predictive value of serum vaspin level in the amelioration of fatty liver and metabolic disturbance in patients with severe obesity after LVBG. LVBG = laparoscopic vertical banded gastroplasty, ROC = receiver operating characteristic.

In the ROC curve analysis according to the criteria of obesity (BMI ≥ 35 kg/m^2^) and diagnostic criteria of CT value for fatty liver, patients with BMI ≥35 kg/m^2^ and liver/spleen ratio of CT value ≤1 after surgery were considered to be unimproved in fatty liver and metabolic disturbances, and patients with BMI <35 kg/m^2^ and liver/spleen ratio of CT value >1 after surgery were considered as improved. Then an ROC curve was drawn to analyze the predictive value of serum vaspin level in the amelioration of fatty liver and metabolic disturbance in patients with severe obesity after LVBG (Fig. 2). Area under the curve (AUC) was 0.871 (95% confidence interval [CI] = 0.810–0.931, *P* <.001), which demonstrated that the serum vaspin level has a predictive value for the amelioration of fatty liver and disturbances in metabolism after the surgery is done. And the sensitivities of serum vaspin level ≤0.6, 0.7, 0.8 and 0.9 in predicating the amelioration of fatty liver and metabolic disturbance were 93.90%, 92.68%, 92.68%, and 87.80%, respectively, with the specificities of 28.05%, 35.37%, 51.22%, and 78.05%, respectively, and the accuracies of 63.38%, 66.12%, 73.47%, and 83.28%, respectively. According to the research in the clinical and epidemiological studies the serum vaspin level ≤0.9 is the most common diagnostic criteria for the prevalence of peripheral arterial disease (PAD). Therefore, the serum vaspin level could be used as the cut-off value to predict the amelioration of fatty liver and metabolic disturbance in patients with severe obesity after surgery, among all which had high specificity, good sensitivity, and high accuracy for getting good results relatively.

### 3.7 Logistic regression analysis

Logistics regression is defined as a regression model where the dependent variable (DV) is categorical, and the analysis conducted by this regression is called logistics regression analysis, and it was performed with physical activity, BMI, blood glucose level, FINS, serum vaspin level ≤0.9, and liver enzyme levels such ALT, AST, and γ-GT as independent variables, there are also dependent variables likewise amelioration of fatty liver and metabolic disturbance in the patients with severe obesity after LVGB. The results are shown in Table 5. BMI, FINS, and serum vaspin level ≤0.9 were factors that influenced the amelioration of fatty liver and metabolic disturbance in patients with severe obesity. Their odds ratios (OR) were 3.294 (95%CI = 1.602–6.775, *P* = .001), 1.413 (95%CI = 1.138–1.755, *P* = .002), and 0.009 (95%CI = 0.000–0.272, *P* = .007), respectively. And the partial regression coefficients were 1.192, 0.346, and –4.743, respectively. Additionally, physical activity, blood glucose, ALT, AST, and γ-GT were excluded from predictive factors in the amelioration of fatty liver and metabolic disturbance in patients with severe obesity (*P* >.05).

**Table 5:**
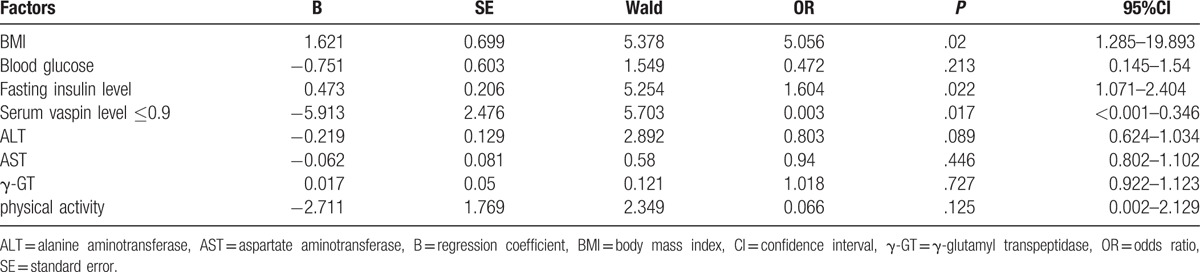
Logistic regression analysis for factors that influence the amelioration of fatty liver and metabolic disturbance in patients with severe obesity.

## 4 Discussion

In the discussion, the most important thing should be discussed is the Vaspin, which is one of the adipokines and associated with obesity,^[26]^ and LVBG is one of the most frequent and effective treatments for morbid obesity.^[27]^ We investigated the role of serum vaspin level in predicting the amelioration of fatty liver and metabolic disturbance in patients with severe obesity following LVBG. Our findings indicate that patients have improved fatty liver and metabolic disturbance after LVBG, and serum vaspin level acts as a predictive indicator of improved fatty liver and metabolic disturbance.

The main thing about this study was found that the condition of metabolic disturbance improved and eventually the serum vaspin level decreased gradually. This may reveal that vaspin could act as a harmful molecule in the development of metabolic disturbances after LVBG. By performing the potential treatment for metabolic disturbance, the excessive amount of VAT is decreased. In the deep cases, it is also proved that there is a positive association between serum vaspin levels and metabolic disturbance in the humans.^[19]^ Dysregulation of adipokines production with anti-inflammatory properties may contribute to metabolic disturbance related to obesity.^[28]^ The vaspin demonstrated by the Phalitakul et al^[29]^ is considered as a novel adipokine, which plays an anti-inflammatory role in vascular smooth muscle cells. Therefore, it is reasonable that vaspin may cause metabolic disturbance with its anti-inflammatory action, especially in abnormal higher level. The at most important thing is serum vaspin has anti-inflammatory property through antioxidative mechanism; this mechanism protects the effects on metabolic diseases.^[30]^ Although further studies are needed, these results suggest that vaspin might be involved in the development of metabolic disturbance. And we suggest an underlying mechanism that low level of vaspin may improve metabolic disturbance by inhibiting its anti-inflammatory property through suppression of antioxidative effect.

Meanwhile, it has also been found that fatty liver increased while serum vaspin level decreased, which revealed a correlation between low level of human serum vaspin and amelioration of fatty liver following LVBG. Orlik et al^[31]^ demonstrated that insulin resistance plays a critical role in the pathogenesis of nonalcoholic fatty liver. Besides, serum vaspin level is closely associated with insulin resistance because fatty liver increased while serum vaspin level decreased.^[32]^ Weight loss affects serum vaspin level in obese people by the regulation of insulin resistance.^[33]^ The findings in this study support our assumption, that there seems to be a strong association between low-expressed vaspin and also fatty liver through the mechanism of insulin resistance, this is the most important thing. And it completely proved that through the signaling pathways, the substrate flux are leading the pathogenesis of insulin resistance.^[34]^ And adipokines regulate various insulin resistance-related pathways through 3 mechanisms: endocrine, paracrine, or autocrine.^[35]^ Thus, it is reasonable to hypothesize that vaspin plays a role in modulating insulin resistance-related signaling pathways of via secretion. Changes in serum vaspin level related to meal might be modulated by insulin rather than by nutrient intake.^[36]^ In contrast, Oberbach et al^[37]^ proved that exercise associated improvement in insulin sensitivity does not help in the reduction of serum vaspin level but oxidative stress is not included. Differences in the results might attribute to diverse period of measurement. Further study with large sample sizes and follow-up should be investigated to warrant our findings.

From the above discussed study, it is clearly proved that the serum vaspin level could be used for predicting the efficacy of LVBG surgery in the severe obesity patients. Furthermore, low serum vaspin levels indicate the amelioration of severe obesity due to weight loss. Few similar changes have been reported not only for amelioration but also for other surgery too. Laparoscopic restrictive bariatric surgery reduced the serum vaspin levels by decreasing dietary intake in a short term.^[38]^ However, elevated serum vaspin level was observed in women with polycystic ovary syndrome in response to weight loss, because a small weight reduction cannot affect serum vaspin levels significantly.^[39]^ Thus, it's proved that the serum vaspin level can be reduced by the patients having large weight reduction. Moreover, in a long term, insulin resistance may not be the reason which caused the change in serum vaspin level after LVBG.

In conclusion, our study provides evidence that serum vaspin level could be regarded as the predictor for improvement of fatty liver and metabolic disturbance after LVBG surgery in patients with severe obesity. However, the exact correlation between changes in serum vaspin level and insulin sensitivity is not clear at early period. The underlying mechanism needs more strong evidence that anti-inflammatory property and antioxidative effect regulated by vaspin might involve in the development of fatty liver and metabolic disturbance. Besides, the number of physical activities of obese patients was small, which may cause certain deviations in the results. Therefore, further study with follow-up is needed to confirm our results.

## Acknowledgment

The authors thank the reviewers for critical comments.

## References

[1] Wang YC McPherson K Marsh T Health and economic burden of the projected obesity trends in the USA and the UK. Lancet 2011;378:815–25. 21872750 10.1016/S0140-6736(11)60814-3

[2] Rurik I Torzsa P Szidor J A public health threat in Hungary: obesity, 2013. BMC Public Health 2014;14:798. 25096526 10.1186/1471-2458-14-798 PMC4143555

[3] Magi R Manning S Yousseif A Contribution of 32 GWAS-identified common variants to severe obesity in European adults referred for bariatric surgery. PLoS One 2013;8:e70735. 23950990 10.1371/journal.pone.0070735 PMC3737377

[4] Finkelstein EA Khavjou OA Thompson H Obesity and severe obesity forecasts through 2030. Am J Prev Med 2012;42:563–70. 22608371 10.1016/j.amepre.2011.10.026

[5] Seetho IW Wilding JP How to approach endocrine assessment in severe obesity? Clin Endocrinol (Oxf) 2013;79:163–7. 23734868 10.1111/cen.12256

[6] Wehr E Pilz S Boehm BO The lipid accumulation product is associated with increased mortality in normal weight postmenopausal women. Obesity (Silver Spring) 2011;1873–80. 21394091 10.1038/oby.2011.42

[7] Robbins JM Mallya G Polansky M Prevalence, disparities, and trends in obesity and severe obesity among students in the Philadelphia, Pennsylvania, school district, 2006–2010. Prev Chronic Dis 2012;9:E145. 22954057 10.5888/pcd9.120118 PMC3475532

[8] O’Connell J Lynch L Cawood TJ The relationship of omental and subcutaneous adipocyte size to metabolic disease in severe obesity. PLoS One 2010;5:e9997. 20376319 10.1371/journal.pone.0009997 PMC2848665

[9] Guenard F Bouchard L Tchernof A DUSP1 gene polymorphisms are associated with obesity-related metabolic complications among severely obese patients and impact on gene methylation and expression. Int J Genomics 2013;2013:609748. 23986905 10.1155/2013/609748 PMC3748404

[10] Inoue J Wada J Teshigawara S The serum vaspin levels are reduced in Japanese chronic hemodialysis patients. BMC Nephrol 2012;13:163. 23206815 10.1186/1471-2369-13-163 PMC3519721

[11] Courcoulas AP Christian NJ Belle SH Weight change and health outcomes at 3 years after bariatric surgery among individuals with severe obesity. JAMA 2013;310:2416–25. 24189773 10.1001/jama.2013.280928 PMC3955952

[12] Kral JG Kava RA Catalano PM Severe obesity: the neglected epidemic. Obes Facts 2012;5:254–69. 22647306 10.1159/000338566

[13] Cura HS Ozdemir HH Demir CF Investigation of vaspin level in patients with acute ischemic stroke. J Stroke Cerebrovasc Dis 2014;23:453–6. 23594688 10.1016/j.jstrokecerebrovasdis.2013.03.023

[14] Hida K Poulsen P Teshigawara S Impact of circulating vaspin levels on metabolic variables in elderly twins. Diabetologia 2012;55:530–2. 22116078 10.1007/s00125-011-2385-0

[15] Kadoglou NP Gkontopoulos A Kapelouzou A Serum levels of vaspin and visfatin in patients with coronary artery disease-Kozani study. Clin Chim Acta 2011;412:48–52. 20850423 10.1016/j.cca.2010.09.012

[16] Bao JP Jiang LF Li J Visceral adipose tissue-derived serine protease inhibitor inhibits interleukin-1beta-induced catabolic and inflammatory responses in murine chondrocytes. Mol Med Rep 2014;10:2191–7. 25118941 10.3892/mmr.2014.2478

[17] Auguet T Quintero Y Riesco D New adipokines vaspin and omentin. Circulating levels and gene expression in adipose tissue from morbidly obese women. BMC Med Genet 2011;12:60. 21526992 10.1186/1471-2350-12-60 PMC3107780

[18] Kukla M Mazur W Buldak RJ Potential role of leptin, adiponectin and three novel adipokines--visfatin, chemerin and vaspin—in chronic hepatitis. Mol Med 2011;17:1397–410. 21738955 10.2119/molmed.2010.00105 PMC3321801

[19] Dimova R Tankova T The role of vaspin in the development of metabolic and glucose tolerance disorders and atherosclerosis. Biomed Res Int 2015;2015:823481. 25945347 10.1155/2015/823481 PMC4402467

[20] Nakatsuka A Wada J Iseda I Vaspin is an adipokine ameliorating ER stress in obesity as a ligand for cell-surface GRP78/MTJ-1 complex. Diabetes 2012;61:2823–32. 22837305 10.2337/db12-0232 PMC3478540

[21] Nakatsuka A Wada J Iseda I Visceral adipose tissue-derived serine proteinase inhibitor inhibits apoptosis of endothelial cells as a ligand for the cell-surface GRP78/voltage-dependent anion channel complex. Circ Res 2013;112:771–80. 23307819 10.1161/CIRCRESAHA.111.300049

[22] Aktas B Yilmaz Y Eren F Serum levels of vaspin, obestatin, and apelin-36 in patients with nonalcoholic fatty liver disease. Metabolism 2011;60:544–9. 20580037 10.1016/j.metabol.2010.05.008

[23] Hemmingsson E Hellenius ML Ekelund U Bergstrom J, and Rossner S. Impact of social support intensity on walking in the severely obese: a randomized clinical trial. Obesity (Silver Spring) 2008;16:1308–13. 18388901 10.1038/oby.2008.204

[24] Tudor-Locke C Bassett DR Jr How many steps/day are enough? Preliminary pedometer indices for public health. Sports Med 2004;34:1–8. 14715035 10.2165/00007256-200434010-00001

[25] Zhou BF Cooperative Meta-Analysis Group of the Working Group on Obesity in C. Predictive values of body mass index and waist circumference for risk factors of certain related diseases in Chinese adults—study on optimal cut-off points of body mass index and waist circumference in Chinese adults. Biomed Environ Sci 2002;15:83–96. 12046553

[26] Ko BJ Lee M Park HS Elevated vaspin and leptin levels are associated with obesity in prepubertal Korean children. Endocr J 2013;60:609–16. 23318644 10.1507/endocrj.ej12-0384

[27] Rebecchi F Rocchietto S Giaccone C Gastroesophageal reflux disease and esophageal motility in morbidly obese patients submitted to laparoscopic adjustable silicone gastric banding or laparoscopic vertical banded gastroplasty. Surg Endosc 2011;25:795–803. 20676689 10.1007/s00464-010-1257-x

[28] Katsareli EA Dedoussis GV Biomarkers in the field of obesity and its related comorbidities. Expert Opin Ther Targets 2014;18:385–401. 24479492 10.1517/14728222.2014.882321

[29] Phalitakul S Okada M Hara Y A novel adipocytokine, vaspin inhibits platelet-derived growth factor-BB-induced migration of vascular smooth muscle cells. Biochem Biophys Res Commun 2012;423:844–9. 22713468 10.1016/j.bbrc.2012.06.052

[30] Kameshima S Sakamoto Y Okada M Vaspin prevents elevation of blood pressure through inhibition of peripheral vascular remodelling in spontaneously hypertensive rats. Acta Physiol (Oxf) 2016;217:120–9. 26640237 10.1111/apha.12636

[31] Orlik B Handzlik G Olszanecka-Glinianowicz M The role of adipokines and insulin resistance in the pathogenesis of nonalcoholic fatty liver disease. Postepy Hig Med Dosw (Online) 2010;64:212–9. 20498498

[32] Teshigawara S Wada J Hida K Serum vaspin concentrations are closely related to insulin resistance, and rs77060950 at SERPINA12 genetically defines distinct group with higher serum levels in Japanese population. J Clin Endocrinol Metab 2012;97:E1202–7. 22539588 10.1210/jc.2011-3297

[33] Chang HM Lee HJ Park HS Effects of weight reduction on serum vaspin concentrations in obese subjects: modification by insulin resistance. Obesity (Silver Spring) 2010;18:2105–10. 20339362 10.1038/oby.2010.60

[34] Samuel VT Shulman GI The pathogenesis of insulin resistance: integrating signaling pathways and substrate flux. J Clin Invest 2016;126:12–22. 26727229 10.1172/JCI77812 PMC4701542

[35] Chang HM Park HS Park CY Association between serum vaspin concentrations and visceral adipose tissue in Korean subjects. Metabolism 2010;59:1276–81. 20060144 10.1016/j.metabol.2009.11.021

[36] Kovacs P Miehle K Sandner B Insulin administration acutely decreases vaspin serum concentrations in humans. Obes Facts 2013;6:86–8. 23466514 10.1159/000348836 PMC5644680

[37] Oberbach A Kirsch K Lehmann S Serum vaspin concentrations are decreased after exercise-induced oxidative stress. Obes Facts 2010;3:328–31. 20975299 10.1159/000321637 PMC6452153

[38] Golpaie A Tajik N Masoudkabir F Short-term effect of weight loss through restrictive bariatric surgery on serum levels of vaspin in morbidly obese subjects. Eur Cytokine Netw 2011;22:181–6. 22266100 10.1684/ecn.2011.0295

[39] Koiou E Tziomalos K Dinas K The effect of weight loss and treatment with metformin on serum vaspin levels in women with polycystic ovary syndrome. Endocr J 2011;58:237–46. 21325745 10.1507/endocrj.k10e-330

